# Sensitivity analysis revealing the effect of modulating ionic mechanisms on calcium dynamics in simulated human heart failure

**DOI:** 10.1371/journal.pone.0187739

**Published:** 2017-11-08

**Authors:** Maria T. Mora, Jose M. Ferrero, Lucia Romero, Beatriz Trenor

**Affiliations:** Centro de Investigación e Innovación en Bioingeniería, Universitat Politècnica de València, Valencia, Spain; University of Debrecen, HUNGARY

## Abstract

Abnormal intracellular Ca^2+^ handling is the major contributor to the depressed cardiac contractility observed in heart failure. The electrophysiological remodeling associated with this pathology alters both the action potential and the Ca^2+^ dynamics, leading to a defective excitation-contraction coupling that ends in mechanical dysfunction. The importance of maintaining a correct intracellular Ca^2+^ concentration requires a better understanding of its regulation by ionic mechanisms. To study the electrical activity and ionic homeostasis of failing myocytes, a modified version of the O’Hara et al. human action potential model was used, including electrophysiological remodeling. The impact of the main ionic transport mechanisms was analyzed using single-parameter sensitivity analyses, the first of which explored the modulation of electrophysiological characteristics related to Ca^2+^ exerted by the remodeled parameters. The second sensitivity analysis compared the potential consequences of modulating individual channel conductivities, as one of the main effects of potential drugs, on Ca^2+^ dynamic properties under both normal conditions and in heart failure. The first analysis revealed the important contribution of the sarcoplasmic reticulum Ca^2+^-ATPase (SERCA) dysfunction to the altered Ca^2+^ homeostasis, with the Na^+^/Ca^2+^ exchanger (NCX) and other Ca^2+^ cycling proteins also playing a significant role. Our results highlight the importance of improving the SR uptake function to increase Ca^2+^ content and restore Ca^2+^ homeostasis and contractility. The second sensitivity analysis highlights the different response of the failing myocyte versus the healthy myocyte to potential pharmacological actions on single channels. The result of modifying the conductances of the remodeled proteins such as SERCA and NCX in heart failure has less impact on Ca^2+^ modulation. These differences should be taken into account when designing drug therapies.

## Introduction

Heart failure (HF), characterized by contractile dysfunction and arrhythmogenesis, is the final stage of many cardiovascular diseases. To understand the mechanisms that lead to these pathological conditions, a large body of research has focused on the electrophysiological changes in failing myocytes. At the cellular level, the hallmarks of HF are a prolongation of the action potential and alterations in ionic concentrations, such as intracellular Na^+^ ([Na^+^]_i_) and Ca^2+^ ([Ca^2+^]_i_), as a consequence of ion channel remodeling [1–4].

As intracellular Ca^2+^ is the main regulator of the cardiac excitation-contraction coupling, mishandling of Ca^2+^ is directly related to the mechanical dysfunction and certain arrhythmias associated with HF. Specifically, the alterations in Ca^2+^ dynamics are a decrease of the systolic peak, an increase of the diastolic level, a prolongation of the Ca^2+^ transient (CaT), and a reduced sarcoplasmic reticulum (SR) Ca^2+^ load [5–7], so that it is clear that restoring normal Ca^2+^ cycling in HF could have beneficial therapeutic effects.

The study of the reduced mechanical performance in failing human myocytes has shown a strong correlation with depressed intracellular Ca^2+^ transients and it has also been related to altered mRNA levels of Ca^2+^-handling proteins [8]. Changes in the expression or activity of Ca^2+^ transport proteins in failing human ventricular myocytes, reviewed elsewhere [9–13], highlight the significant alteration in proteins involved in Ca^2+^ removal from the cytosol, such as the sarcoplasmic reticulum Ca^2+^-ATPase (SERCA) and the Na^+^/Ca^2+^ exchanger (NCX), and the existence of an important impaired diastolic Ca^2+^ release from the SR. On this basis, therapies are now being designed to restore Ca^2+^ homeostasis by targeting Ca^2+^-handling proteins [14,15]. Most of these consist of improving SR function by increasing SR Ca^2+^ uptake or preventing SR Ca^2+^ leak, although they have not been systematically explored. There are also other ionic transporters involved in the electrophysiology of the heart that have not been analyzed from this point of view.

Pharmacological agents interacting with specific ionic channels can improve the electrical and mechanical properties of the heart. In fact, many studies have focused on the pharmacological effects on the action potential, but less attention has been paid to the effects on Ca^2+^ dynamics. The need for a better understanding of the mechanisms in this complex electrophysiological system requires a systematic methodology. Mathematical models and computational simulations can help identify and explain interactions and effects that cannot be easily understood experimentally. In cardiac electrophysiology, studies using this approach have provided new findings on the ionic basis of arrhythmogenic processes [16,17], while electromechanical models have highlighted the need for a balanced Ca^2+^ handling mechanism to prevent cardiac dysfunction, since treatments targeting increased contractility alone are not sufficient [18].

The aim of this study is thus to analyze *in silico* the alterations of Ca^2+^ handling in failing human myocytes and to identify possible pharmacological targets that could restore them. The first sensitivity analysis, performed in a HF model, elucidates the main mechanisms responsible for the alterations of Ca^2+^ homeostasis in such pathological conditions. Secondly, the comparison of two sensitivity analyses, in normal and failing conditions, reveals differences between both models in the effect of ionic parameters on Ca^2+^ handling. These findings suggest that pharmacological treatments might not produce the same results in HF as in healthy myocytes, meaning that it may be necessary to adapt the treatment to the specific pathological situation.

## Methods

### Human action potential model

**Fig 1:**
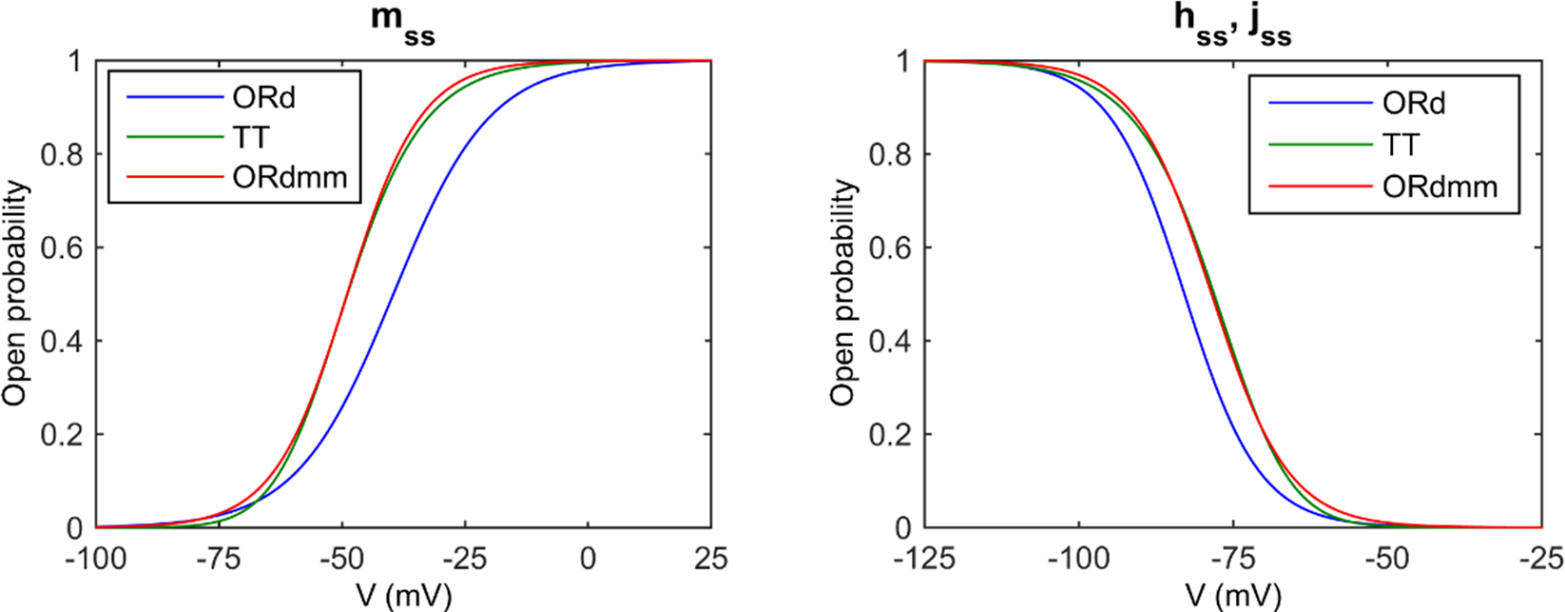
Steady state activation (Left) and inactivation (Right) gates of the fast Na^+^ current in the different models. Original O’Hara et al. model (ORd), ten Tusscher et al. model (TT), and the modified ORd model (ORdmm).

The most recent and complete human ventricular action potential (AP) model is that of O’Hara et al. (ORd) [19], which provides a detailed description of Ca^2+^ handling, allowing the analysis of electrophysiological characteristics related to Ca^2+^. A modified version of this model was used to simulate the electrophysiological activity at the cellular level. The reason for not using the original ORd formulation is that in a 1D multicellular fiber, conduction velocity is low, especially under pathological situations when sodium current is reduced. Thus, the original fast sodium current (I_Na_) formulation was modified by shifting its steady state activation (m_ss_) and inactivation gates (h_ss_ and j_ss_), as depicted in Fig 1. Passini et al. [20] had already proposed an optimized formulation for the sodium steady-state inactivation response, which consisted basically of matching ten Tusscher et al. [21] curves by shifting the half potential and changing the slope. The m_ss_ was left-shifted, as suggested by Passini et al. [20], but we also modified the slope of the curve. The conductance (G_Na_) was reduced by 60% to maintain (dV/dt)_max_ in the range of 260 V/s.

Since it has been reported in voltage-clamp experiments [22] that the late sodium current (I_NaL_) is 0.07% of the value of I_Na_ peak at -30 mV after 200 ms, its conductance value (G_NaL_) was doubled to satisfy this condition. From now on, we will refer to the ORd modified model as the ORdmm.

### Simulation protocol

**Fig 2:**
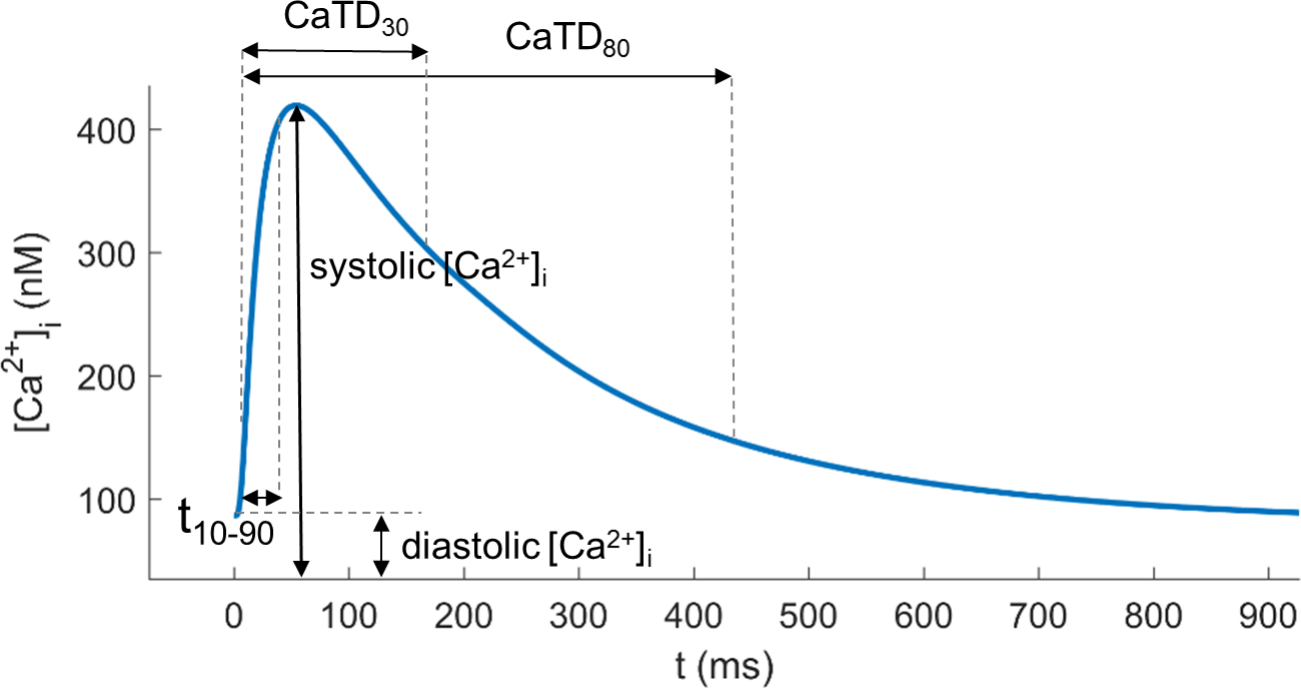
Time course of a steady state Ca^2+^ transient (CaT) and its electrophysiological characteristics. Systolic [Ca^2+^]_i_ and diastolic [Ca^2+^]_i_, CaT duration measured as the time from upstroke to 30% and 80% recovery (CaTD_30_ and CaTD_80_) and 10% to 90% CaT rise time (t_10-90_).

All the simulations in this study were carried out in endocardial cells paced at 1 Hz. Quantitative indicators characterizing Ca^2+^ dynamics were measured for the last of 1000 beats, after steady-state was reached (Figs 1 and 2 of the S1 File show the last traces of AP and CaT in different simulations). The measured electrophysiological (EP) indicators were: systolic peak and diastolic value of [Ca^2+^]_i_, Ca^2+^ transient (CaT) duration measured as the time from upstroke to 30% and 80% recovery (CaTD_30_ and CaTD_80_), rise time of CaT (t_10-90_) defined as the time from 10% CaT (close to the baseline) to 90% CaT (close to peak) (see Fig 2 for details). Other important concentrations are systolic and diastolic values of Ca^2+^ reached in the subspace ([Ca^2+^]_ss_), representing the space near the T-tubules, and in the sarcoplasmic reticulum (SR), which is divided into the junctional (JSR) and network SR (NSR). AP duration (APD) was measured as the time from the upstroke to 30% and 90% repolarization (APD_30_ and APD_90_), as well as the [Na^+^]_i_ peak.

### Sensitivity analysis of the HF model

An AP model for the failing myocyte was formulated on the basis of experimental observations to reproduce the main EP characteristics under these conditions in failing human ventricular myocytes. Table 1 shows the changes performed in the ORdmm model, following Gomez et al. changes performed in the original ORd model [23]. This electrical remodeling consists of applying a scale factor to maximal conductances of the I_NaL_, the transient outward K^+^ current (I_to_), and the inward rectifier K^+^ current (I_K1_), to the time constant of inactivation of the I_NaL_ (τ_hL_), to the maximal fluxes of the Na^+^/ K^+^ ATPase current (I_NaK_), the Na^+^/Ca^2+^ exchange current (I_NCX_), the Ca^2+^ uptake via SERCA pump (J_SERCA_), and the SR Ca^2+^ leak (J_leak_), to the fraction of active binding sites of the Ca^+2^ calmodulin-dependent protein kinase II (CaMKa), and to the SR Ca^2+^-dependence of the steady-state activation of ryanodine receptor (RyR) release (K_rel,Ca_). Further details of these variables can be found in the Supplementary Material (S1 File).

**Table 1:** HF remodeling in ORdmm model.

Ionic parameter	% in the HF model compared to the ORdmm
I_NaL_	180%
τ_hL_	180%
I_to_	40%
I_K1_	68%
I_NaK_	70%
I_NCX_	175%
CaMKa	150%
J_SERCA_	50%
J_leak_	130%
K_rel,Ca_	80%

The modified parameters are: the late Na^+^ current (I_NaL_), the time constant of inactivation of the I_NaL_ (τ_hL_), the transient outward current (I_to_), the inward rectifier K^+^ current (I_K1_), the Na^+^/K^+^ pump current (I_NaK_), the Na^+^/Ca^2+^ exchanger (I_NCX_), the fraction of active binding sites of the Ca^+2^ calmodulin-dependent protein kinase II (CaMKa), the sarcoplasmic reticulum (SR) Ca^2+^ pump (J_SERCA_), the SR Ca^2+^ leak (J_leak_) and the sensitivity to [Ca^2+^]_JSR_ of the ryanodine receptors (Ca ^2+^ sensitivity of J_rel,∞_, called K_rel,Ca_).

A sensitivity analysis was performed to study the variability of HF remodeling. The ionic parameters altered in HF were varied one at a time with a new scale factor to assess the impact of their variability on Ca^2+^ dynamics. The baseline model was the ORdmm model with the EP remodeling described in Table 1 (“HF basic”). Different degrees of HF were evaluated: without HF (“no change”), intermediate HF (“50% HF”) and severe HF (“150% HF”), and applied to one parameter at a time in each simulation, as detailed below. First, each remodeled variable was individually modified to its normal value as in the ORdmm model, i.e. without HF, while the others were fixed to the values of the basic HF model. This gave rise to 10 different “no change” models, each one for a specific parameter. The same parameters were similarly varied one at a time to a value representing ±50% of that observed in the HF basic remodeling. In this way we obtained 10 different basic HF models in which only one parameter was at 50% of its HF condition (“50% HF”) and another 10 in which one parameter was increased by 50% of the basic HF remodeling (“150% HF”) (the exact percentages of change are specified in Table 1 in the S1 File). The different sensitivities of the most severe HF condition (“150% HF”) and the “no change” condition were then calculated, as described in Trenor et al. [17], where the indexes’ percentage of change (D_c,p,x_) and sensitivities (S_c,p_) were calculated as follows:

$$D_{c,p,x}=\frac{c_{p,x}-c_{basic}}{c_{basic}}\cdot 100$$ (1)

$$S_{c,p}=\frac{D_{c,p,2}-D_{c,p,1}}{\Delta a}$$ (2)

with c_p,x_ being the magnitude of the characteristic “c” when parameter “p” undergoes a change with respect to the basic HF model (x = 1: without HF and x = 2: 150% HF), and c_basic_ the value of the same property in the basic HF model; Δa is the total interval of change of parameter p. The evaluated EP characteristics are the indicators specified in the simulation protocol section and were obtained from the steady-state APs and CaTs (see Figure A in S1 File). Finally, the calculated sensitivities were normalized to the maximum absolute sensitivity for each particular characteristic to facilitate the detection of the strongest effects.

### Sensitivity analysis of the effects of potential drugs

One of the aims of the present work is to study how potential drug-induced alterations in the main ionic currents could modulate important electrophysiological characteristics related to Ca^2+^ handling in normal and failing hearts. The methodology employed consisted of modulating individual ionic conductances or maximal fluxes by applying a scale factor of ±60%. These variations were considered as a surrogate for enhancement or inhibition of one ionic transport mechanism due to the possible effect of a drug. The targeted currents or fluxes were the main parameters of the model, well known in the EP activity of myocytes: I_Na_, I_NaL_, I_to_, the L-type Ca^2+^ current (I_CaL_), the rapid delayed rectifier K^+^ current (I_Kr_), the slow delayed rectifier K^+^ current (I_Ks_), I_K1_, I_NCX_, I_NaK_, J_SERCA_, the SR Ca^2+^ release flux via RyR (J_rel_), J_leak_, and the Na^+^ background current (I_Nab_). Since we wanted to study and compare the modulation in normal and failing conditions, these changes were applied to both models separately, obtaining two sensitivity analyses. When the HF model was used, modifications were done by maintaining the electrical remodeling affecting ionic parameters. Once the simulations had been performed, the EP characteristics were measured from the steady-state APs and CaTs (see Figure B in S1 File). For each EP characteristic and parameter, the indexes’ percentage of change (D_c,p,x_) and sensitivities (S_c,p_) were calculated similarly to those of the first sensitivity analysis (Eqs.1 and 2). In this case, there were two basic models, the ORdmm model with and without HF, and sensitivities were calculated from the variation -60% (x = 1) to +60% (x = 2); Δa remains constant and has a value of 1.2.

In the last step, to identify the parameters with the strongest influence on each EP indicator, the relative sensitivity was calculated as the ratio between each sensitivity and the maximum absolute sensitivity for that particular characteristic.

## Results

### Impact of HF remodeling variability on Ca^2+^ dynamics

**Fig 3:**
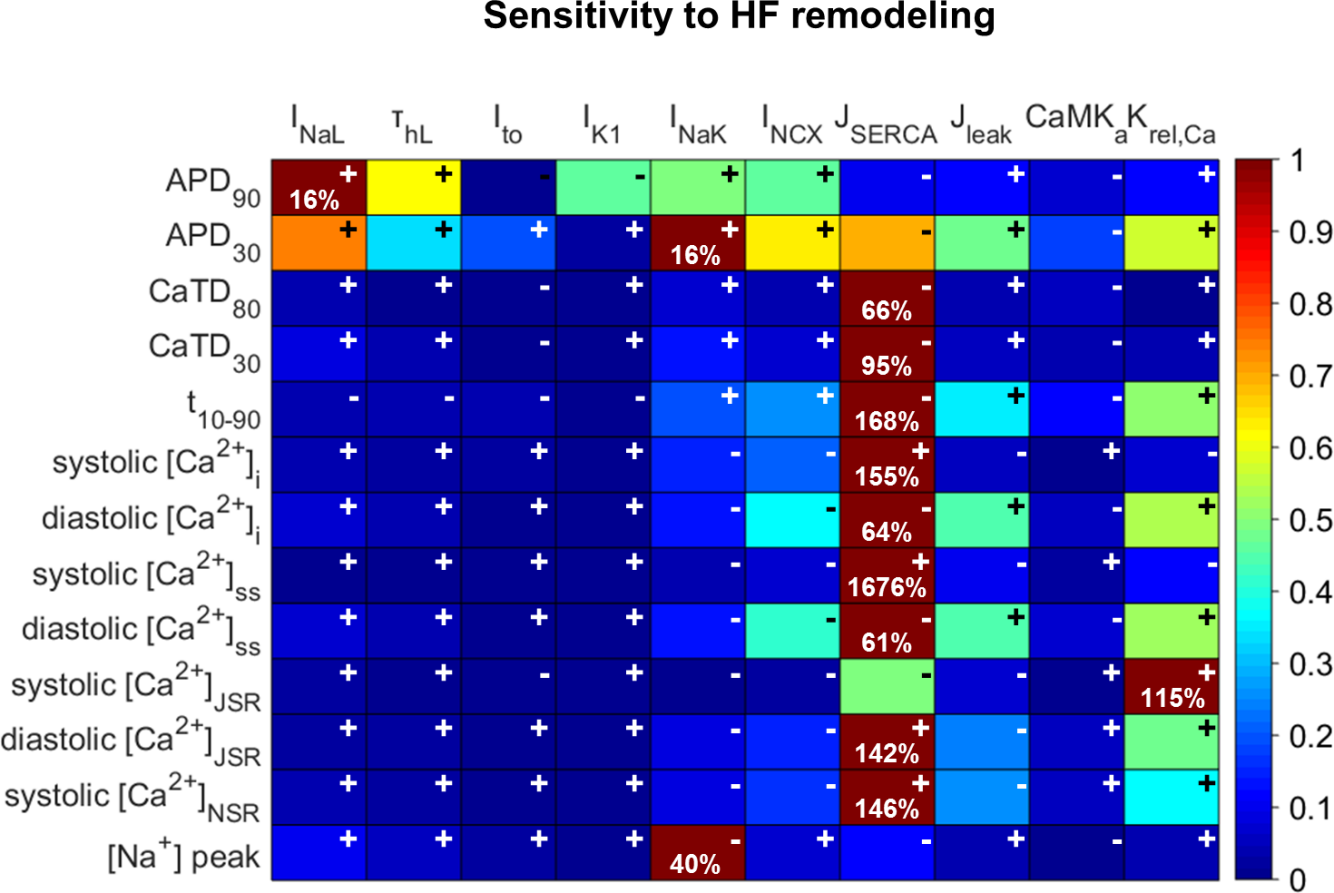
Relative sensitivities of the electrophysiological properties to changes in electrophysiological remodeling in the HF model. In the color scale, dark red indicates maximum relative sensitivity of a particular electrophysiological property to one ionic parameter, whereas dark blue indicates lack of dependency. Signs indicate whether the dependency is direct (+) or inverse (-). Percentages in each box indicate the maximum absolute sensitivity of the EP property in that row to one of the ionic parameters. The modulated parameters are: the late Na^+^ current (I_NaL_), the time constant of inactivation of the I_NaL_ (τ_hL_), the transient outward current (I_to_), the inward rectifier K^+^ current (I_K1_), the Na^+^/K^+^ pump current (I_NaK_), the Na^+^/Ca^2+^ exchanger (I_NCX_), the fraction of active binding sites of the Ca^+2^ calmodulin-dependent protein kinase II (CaMKa), the sarcoplasmic reticulum (SR) Ca^2+^ pump (J_SERCA_), the SR Ca^2+^ leak (J_leak_) and the sensitivity to [Ca^2+^]_JSR_ of the RyR (Ca^2+^ sensitivity of J_rel,∞_, called K_rel,Ca_). The electrophysiological properties are: action potential duration (APD_90_ and APD_30_), Ca^2+^ transient duration (CaTD_80_ and CaTD_30_), rise time of CaT (t_10-90_), systolic and diastolic Ca^2+^ levels in the cytosol ([Ca^2+^]_i_), the subsarcolemmal space ([Ca^2+^]_ss_), the junctional SR ([Ca^2+^]_JSR_) and the network SR ([Ca^2+^]_NSR_), and intracellular Na^+^ peak ([Na^+^]_i_).

Firstly, we analyzed the individual effects of the variability on the different ionic parameters remodeled in HF on Ca^2+^ indicators (see Fig 3), to find the relative sensitivities of all the combinations of EP characteristics (vertical) to parameter variations (horizontal). A color scale, from dark blue to dark red highlights the strongest effects of each indicator (rows). The maximum absolute sensitivity (S_c,p_) is shown in each row to indicate the total impact of the parameters on a particular characteristic. In addition to sensitivity values, it is also important to find the dependency directions between the variables. For this, positive and negative signs indicate whether the change of the ionic current and the HF characteristic follow the same tendency or an inverse tendency, respectively.

**Fig 4:**
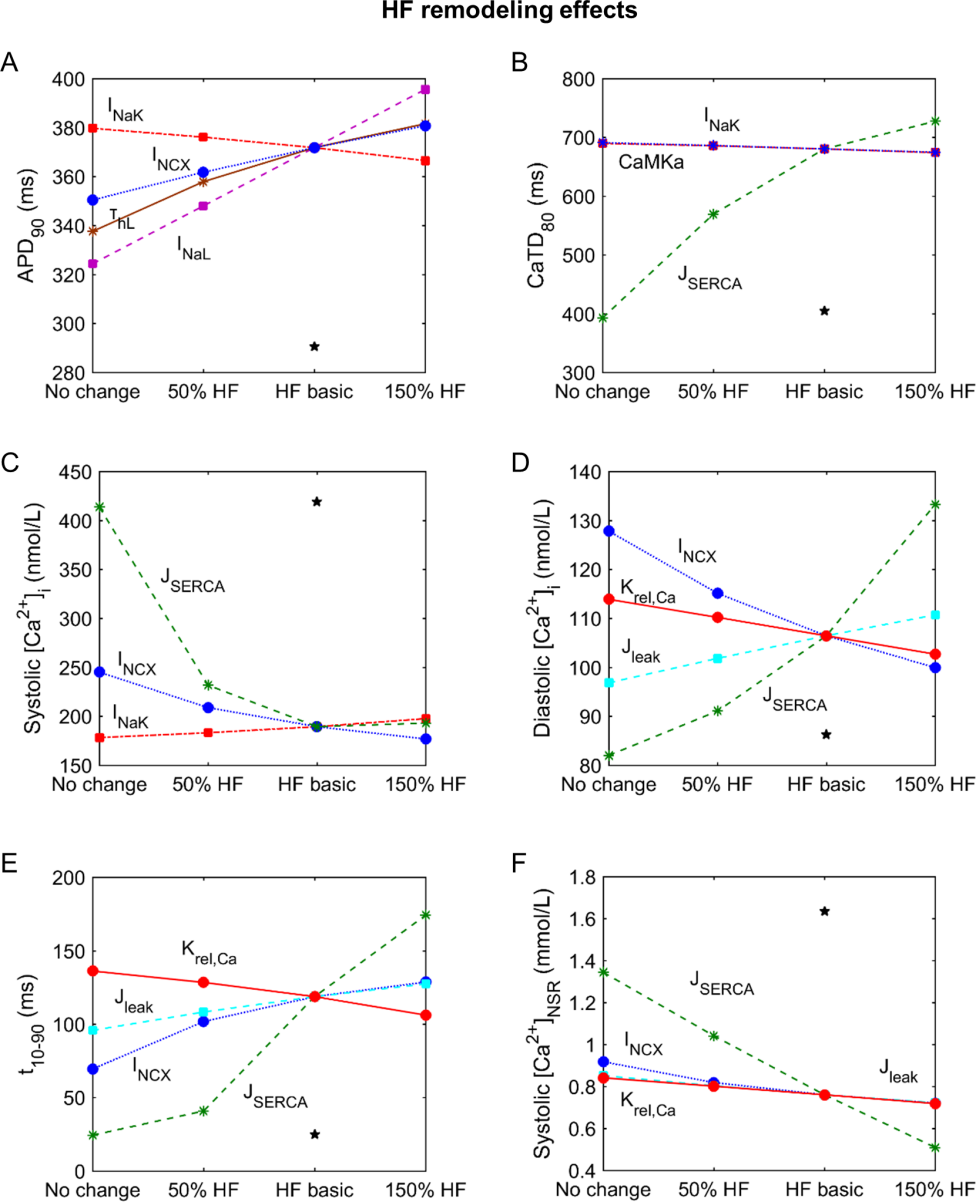
Sensitivity of electrophysiological properties to changes in ionic current parameters in the HF model. Changes in APD_90_ (panel A), CaTD_80_ (panel B), systolic and diastolic [Ca^2+^]_i_ (panels C and D), rise time of CaT (panel E) and SR Ca^2+^systolic load (panel F) with the ionic parameters labeled. Axis x represents the simulation conditions; for ‘‘HF basic” the remodeling of the basic HF model is considered, for ‘‘No change” the labeled parameter is unchanged as it is in the ORdmm model, for ‘‘50% HF” the HF condition is reduced by 50% in the labeled parameter and for ‘‘150% HF” the HF condition is increased by 50%. The star represents the value of the indicator under simulated normal conditions.

From this sensitivity analysis, it can be deduced that I_NaL_ and I_NaK_ are the most important contributors to APD variations. The enhancement of both currents prolongs APD, but the former has a greater effect on APD_90_ and the latter on APD_30_. τ_hL_ and I_NCX_ also have a mild effect. Fig 4 shows the values of selected EP indicators with the variations of the parameters and the currents that exert a relevant influence. Panel A shows how APD_90_ is modulated by these parameters.

**Fig 5:**
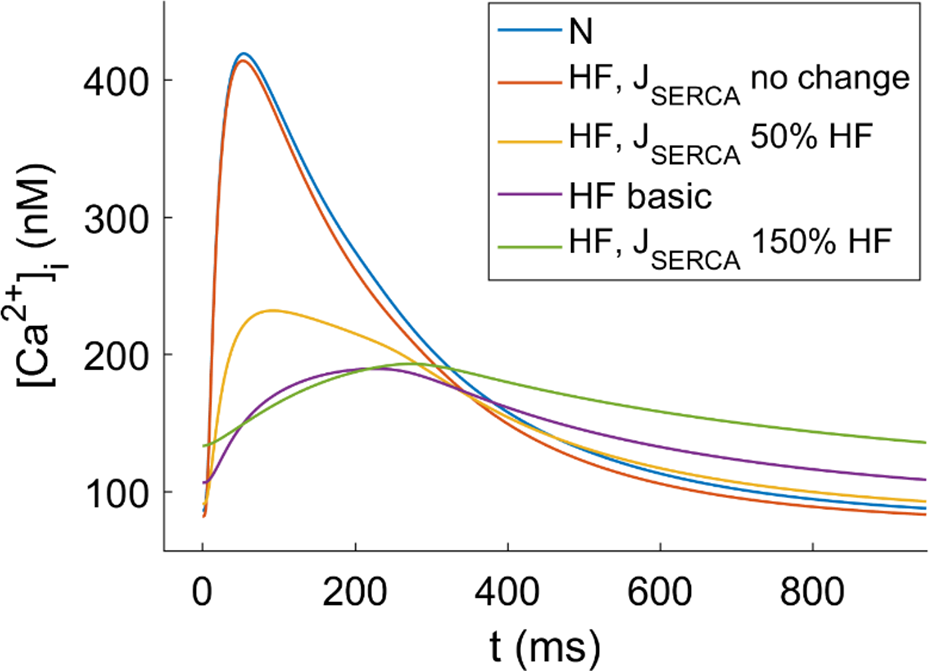
Effect of J_SERCA_ remodeling in HF on CaTs. Normal (N) vs failing (HF) conditions varying J_SERCA_ from the original value in the ORdmm model (no change) to 150% of its value in HF.

Interestingly, J_SERCA_ is the major contributor to most of the EP properties related to Ca^2+^ dynamics (Fig 3). It can be seen in Fig 4 that the SERCA “no change” condition is able to restore these indicators to their normal values, despite EP remodeling in other parameters due to HF. Fig 5 shows the changes in CaT morphology due to SERCA remodeling in HF; a considerable recovery can be obtained by simply restoring the SR Ca^2+^ uptake function. CaTD shows a negative sensitivity to SERCA and the duration is increased with the inhibition of the pump during HF (Fig 4B, dashed green line). The other ionic parameters with an effect on CaTD are far from the influence of J_SERCA_. For instance, although the reduction of I_NaK_ (dashed red line) has a positive dependence and could help to reduce the duration, the sensitivity value is small. Thus, restoring J_SERCA_ to its normal value would restore the CaTD value of non-failing myocytes (around 400 ms). The CaTD_30_ sensitivities show a similar tendency to the CaTD_80_ sensitivities in all the parameters but the values are higher.

Another important characteristic of Ca^2+^ dynamics is the rise time (t_10-90_). The SERCA pump is the strongest modulator of t_10-90_. As shown in Fig 3, J_leak_ and K_rel,Ca_ also affect t_10-90_, although to a lesser extent than SERCA_._ Both parameters are related with Ca^2+^ extrusion from the SR, in the cytosol and the subspace, respectively. Indeed K_rel,Ca_ is the sensitivity of J_rel_ to [Ca^2+^]_JSR_, meaning that a reduction of K_rel,Ca_ leads to a higher release through RyRs. In general, HF conditions slow down the CaT rise, but reduced K_rel,Ca_ helps to accelerate it (Fig 4E, solid red line). The high impact of the abnormal SERCA function, increasing t_10-90,_ is one of the major problems in HF, and the enhancement of I_NCX_ (dotted blue line) contributes to a slow rise in CaT, although to a lesser extent.

[Ca^2+^] in all cell compartments is strongly modulated by the reduced J_SERCA_ in HF. Specifically, systolic [Ca^2+^]_i_ values decrease when SERCA activity is reduced (Fig 4C), while diastolic concentrations increase (Fig 4D). The influence of I_NCX_ (dotted blue line) enhancement is also important and contributes to a general reduction of [Ca^2+^]_i_. Reduced I_NaK_ (dashed red line) also leads to higher systolic Ca^2+^. Diastolic values are also sensitive to changes in J_leak_ and K_rel,Ca_ (dashed blue and solid red lines, respectively). In the subspace, the sensitivity of [Ca^2+^] to ionic parameters is similar to the sensitivity in the cytoplasm, but with higher sensitivity values of the systolic Ca^2+^ peak (see rows 8 and 9 in Fig 3).

As the SR Ca^2+^ load is altered in HF and contributes to CaT changes, Ca^2+^ concentrations during an AP in this compartment were also analyzed. Both the junction and the network compartments have similar sensitivities to EP remodeling changes. In fact, J_SERCA_ (dashed green line) remains the most important factor in modulating SR Ca^2+^ load, followed by J_leak_, K_rel,Ca_, and I_NCX_ (Fig 4F). Exceptionally, it can be seen that in the JSR, K_rel,Ca_ is the main parameter that affects systolic concentration, closely followed by J_SERCA_ (see row 10 in Fig 3). The negative effect of J_SERCA_ on systolic [Ca^2+^]_JSR_ is because of the smaller gradient between intracellular and SR [Ca^2+^], leading to lower J_rel_. Consequently, the systolic [Ca^2+^]_JSR_ minimum peak is less marked and the value becomes more positive as SERCA activity decreases.

The [Na^+^]_i_ analysis shows high dependence on I_NaK_ in an inverse mode, as expected (Fig 3, last row), while the sensitivity of the rest of the parameters is less than 10%.

### Sensitivity of the normal and the HF model to potential effects of drugs

**Fig 6:**
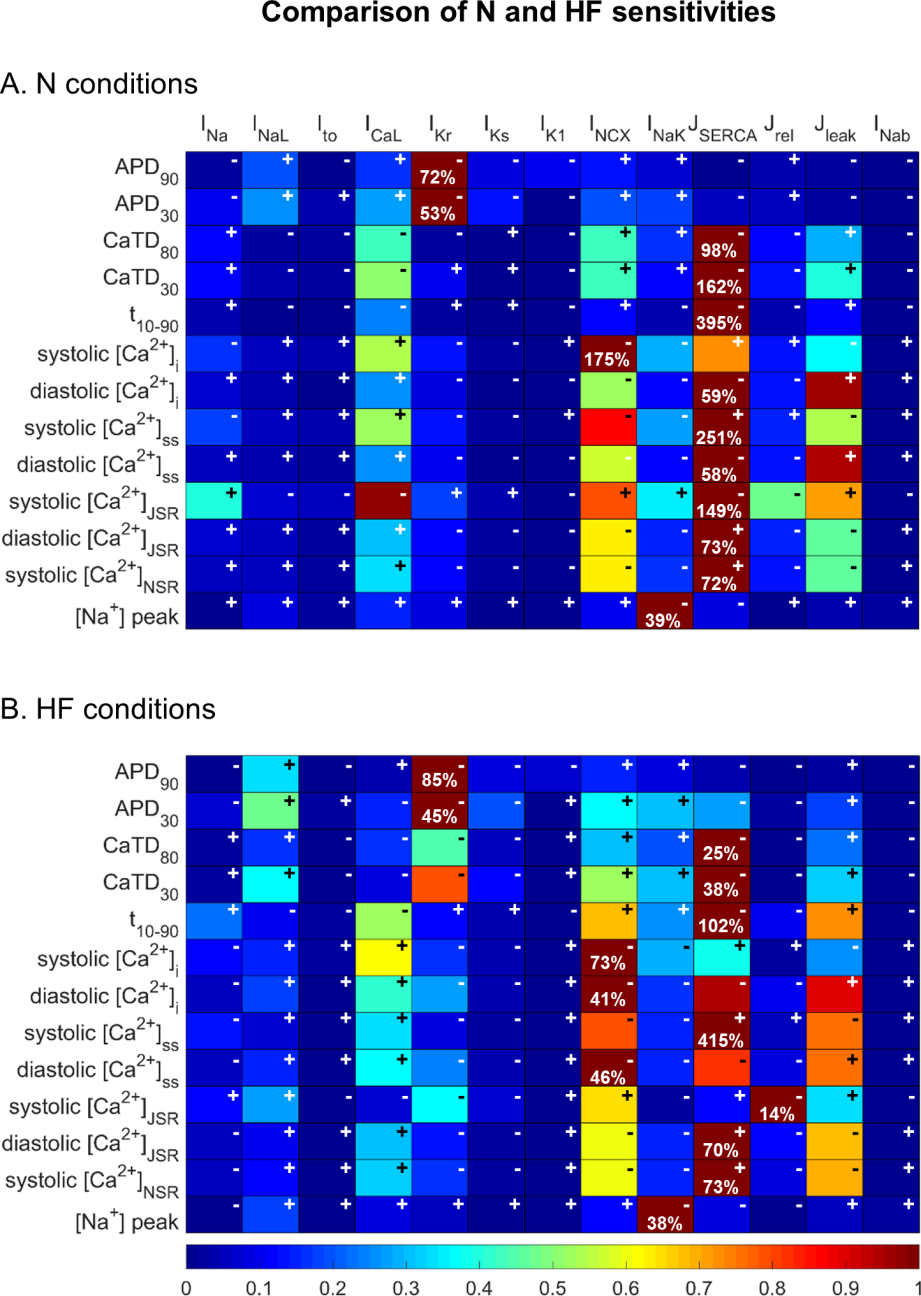
Relative sensitivities of the electrophysiological properties to modulating effects of potential drugs. **Sensitivities in normal (panel A) and failing conditions (panel B).** In the color scale, dark red indicates maximum relative sensitivity of a particular electrophysiological property to one ionic parameter, whereas dark blue indicates lack of dependency. Signs indicate whether the dependency is direct (+) or inverse (-). Percentages in each box indicate the maximum absolute sensitivity of the EP property in that row for all ionic parameters. The modulated parameters are: the fast Na^+^ current (I_Na_,), the late Na^+^ current (I_NaL_), the transient outward K^+^ current (I_to_), the L-type Ca^2+^ current (I_CaL_), the rapid delayed rectifier K^+^ current (I_Kr_), the slow delayed rectifier K^+^ current (I_Ks_), the inward rectifier K^+^ current (I_K1_), the Na^+^/Ca^2+^ exchange current (I_NCX_), the Na^+^/K^+^ pump current (I_NaK_), the Ca^2+^ uptake via SERCA pump (J_SERCA_), the SR Ca^2+^ release flux via RyR (J_rel_), the SR Ca^2+^ leak (J_leak_) and the Na^+^ background current (I_Nab_). The electrophysiological properties are: action potential duration (APD_90_ and APD_30_), Ca^2+^ transient duration (CaTD_80_ and CaTD_30_), rise time of CaT (t_10-90_), systolic and diastolic Ca^2+^ levels in the cytosol ([Ca^2+^]_i_), the subsarcolemmal space ([Ca^2+^]_ss_), the junctional SR ([Ca^2+^]_JSR_) and the network SR ([Ca^2+^]_NSR_), and intracellular Na^+^ peak ([Na^+^]_i_).

Fig 6 summarizes the results of the sensitivity analyses of AP and Ca^2+^ indicators to the same ionic modulation, simulating potential effects of pharmacological treatments in normal and failing conditions, showing the relative sensitivities on a color scale. The differences between the two models are indicated by the colors as well as by the different maximal sensitivities.

**Fig 7:**
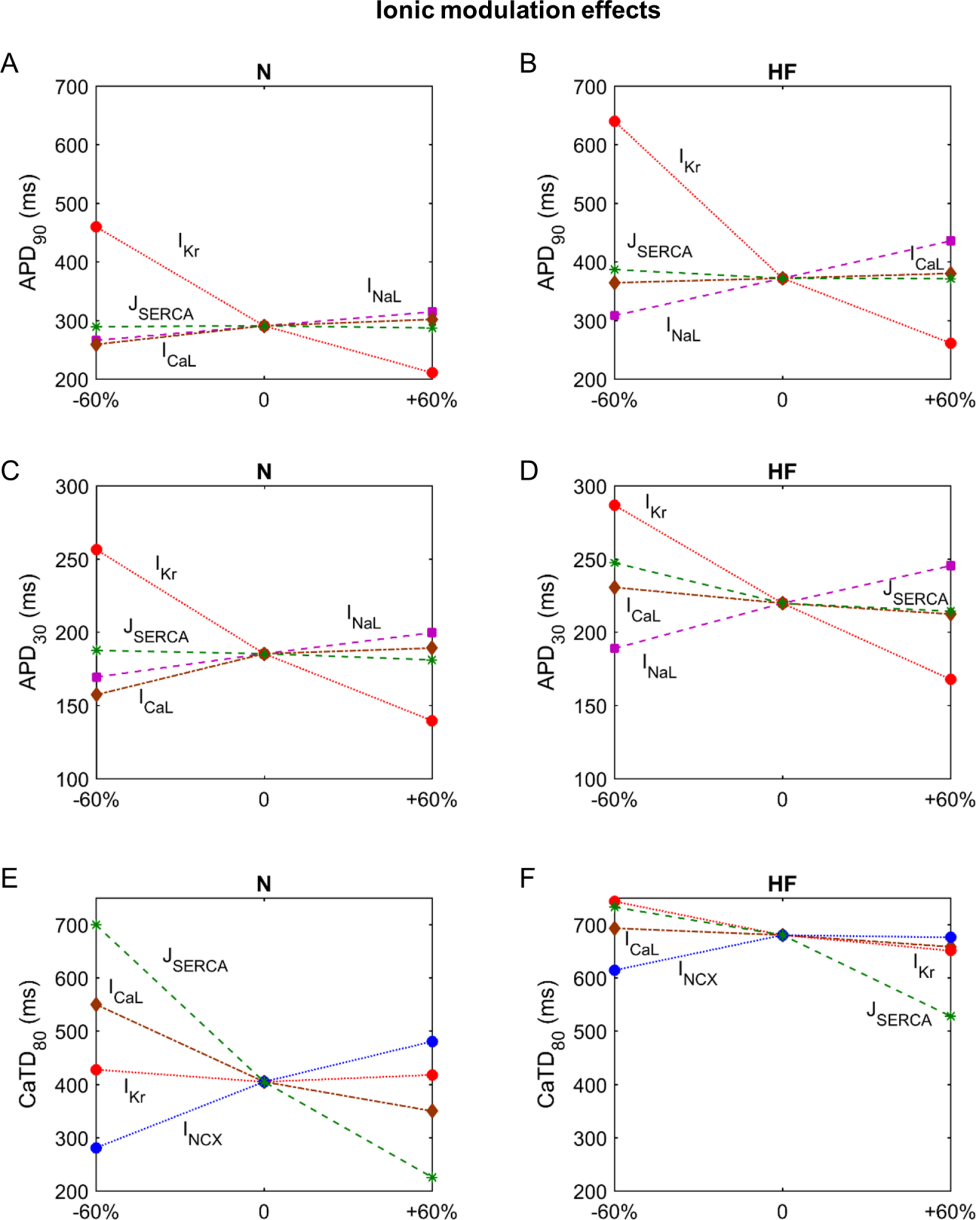
Sensitivity of APD_90_, APD_30_ and CaTD_80_ to the effects of drugs in normal (N) and failing (HF) conditions. Changes in APD_90_ (panels A and B), APD_30_ (panels C and D) and CaTD_80_ (panels E and F). Axis x represents simulation conditions; for ‘‘0” the basic model is considered, for ‘‘± 60%” the labeled parameter has been changed by this percentage.

**Fig 8:**
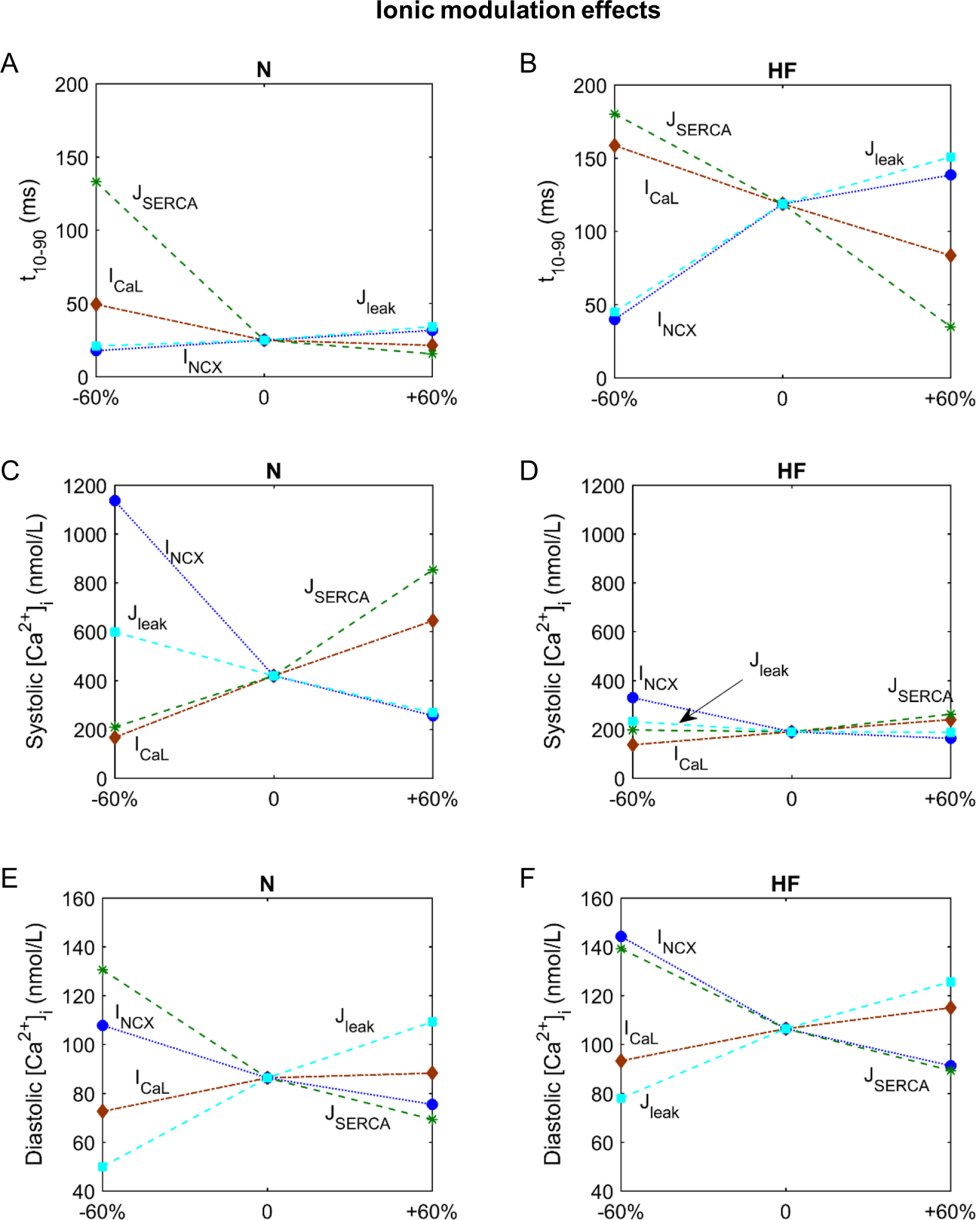
Sensitivity of t_10-90_, systolic and diastolic [Ca^2+^]_i_ to the effects of drugs in normal (N) and failing (HF) conditions. Changes in the rise time of CaT (panels A and B), in systolic and diastolic [Ca^2+^]_i_ (panels C, D and E, F). Axis x represents simulation conditions; for ‘‘0” the basic model is considered, for ‘‘±60%” the labeled parameter has been changed by this percentage.

The response of normal and failing EP properties to the same changes in the main ionic currents can be seen in this sensitivity analysis. In general, the effects on EP characteristics follow the same tendency in both models, but with different sensitivities in some cases, showing a different response to the same change. Figs 7 and 8 show the most important ionic factors leading to important changes in the different indicators (APD_90_, APD_30_ and CaTD_80_ in Fig 7, and t_10-90_ and systolic and diastolic [Ca^2+^]_i_ in Fig 8) in normal and failing myocytes.

First, APD is highly sensitive to I_Kr_, which shortens APD when the current is enhanced (Fig 6, rows 1 and 2 of both panels). I_NaL_ and I_CaL_ are also important contributors to APD changes. However, in comparison with normal conditions, in HF APD_90_ is more sensitive to changes in I_NaL_(compare panels A and B of Fig 7_,_ dashed magenta line). Furthermore, APD_30_ shows a negative dependency on I_CaL_ (dashed brown line) and J_SERCA_ (dashed green line) when these are reduced, which is not seen in normal conditions (Fig 7_,_ panels C and D). Thus, under HF conditions, the I_NaL_ block should be more effective in reducing APD_90_ than in normal conditions, and unexpectedly, an I_CaL_ block or a reduction in J_SERCA_ could have negative effects and increase APD_30_.

With respect to CaT characteristics in normal conditions, again J_SERCA_ seems to be the major contributor (Fig 6, rows 3–7 of both panels). CaTD has a negative sensitivity to SERCA, and although in HF this ionic transport also has a strong effect, it is less marked than in normal conditions. It can be observed that increasing the J_SERCA_ block in the HF model barely increases CaTD, compared to the lengthening produced in normal conditions (Fig 7_,_ panels E and F, dashed green line). However, the enhancement of Ca^2+^ uptake reduces CaTD similarly in both conditions. Blocking NCX (dotted blue line) shortens CaTD, but the influence of I_CaL_ (dashed brown line) is reduced in HF in such a way that an enhancement of this current does not improve CaTD. On the other hand, it is to be noted that the effect of changes in I_Kr_ on CaTD in the HF model becomes important (Fig 6, row 4, panel B). I_Kr_ (dotted red line) block in HF prolongs CaTD, whereas this effect is non-existent in normal conditions. Thus, I_Kr_ block in HF could have negative effects on CaTD, in addition to the prolongation of APD.

Regarding CaT rise time (t_10-90_), a J_SERCA_ block in normal conditions significantly increases this property, acting as the most influential parameter. On the other hand, when it is enhanced, a very slight reduction of t_10-90_ is observed (Fig 8A, dashed green line). However, in HF, SERCA enhancement significantly contributes to the reduction of t_10-90_, reaching a value close to the normal rise time (Fig 8B). Similar reductions can be obtained with the I_NCX_ block (dotted blue line). I_CaL_ and J_leak_ also modulate the rise time, especially in HF.

[Ca^2+^] in all cell compartments is highly regulated by J_SERCA_, followed by I_NCX_, J_leak_, and I_CaL_ (Fig 6, rows 6–12). J_SERCA_ enhancement contributes to the increase of systolic levels and Ca^2+^ SR load and to the reduction of diastolic levels. However, I_NCX_ inhibition increases Ca^2+^ concentration and has a greater effect than SERCA during systole (Fig 6, row 6). In HF, these parameters have less impact on the Ca^2+^ peak and the modification of I_NCX_ (dotted blue line) and J_SERCA_ (dashed green line) produce smaller variations than those in normal conditions (Fig 8, panels C and D). Diastolic [Ca^2+^]_i_ presents a similar sensitivity to I_NCX_ and J_SERCA_ in HF and normal conditions. However, the I_NCX_ block (dotted blue line) has a relevant effect, enhancing diastolic levels and worsening HF changes (Fig 8, panels E and F). In the subspace, responses to variations in ionic currents are similar to those of the cytosol but with higher sensitivity values during the systole (Fig 6, rows 8 and 9).

Ca^2+^ concentrations in the SR are also regulated by J_SERCA_, followed by J_leak_ and I_NCX_. J_rel_ seems to affect only systolic [Ca^2+^]_JSR_ in HF, whereas in normal conditions it is a secondary influence (Fig 6, rows 10–12).

Finally, the [Na^+^]_i_ analysis shows high sensitivity to I_NaK_ in an inverse mode. There are no important differences between normal and HF sensitivities, but I_NaL_ has a higher impact in HF (Fig 6, last row).

## Discussion

### Major findings

In this study Ca^2+^ dynamics and APs were simulated in a human ventricular model (ORdmm) to shed light on the role of some ionic current characteristics and reveal other mechanisms that may take part in Ca^2+^ cycling during HF. The first sensitivity analysis highlights the strong effect of J_SERCA_, among other parameters remodeled in HF, on the modulation of Ca^2+^ indicators. Under HF conditions, depressed SERCA activity worsens all the EP characteristics related to Ca^2+^, which are the hallmark of HF: CaT rise time, systolic and diastolic [Ca^2+^], CaT duration and SR Ca^2+^ content. Enhancing the SERCA uptake function to its normal activity improves all these Ca^2+^ characteristics and could restore contraction.

The comparison of Ca^2+^ sensitivities to drug-induced alterations in the main ionic currents in normal and HF conditions reveals some parameters that modulate Ca^2+^ dynamics in a different way. As blocking I_Kr_ has a marked influence in prolonging CaTD in HF in comparison to its effect in normal conditions, I_Kr_ blocking drugs should be avoided in HF for their impact not only on APD but also on contractility. Blocking I_NCX_ could be beneficial in HF, as it improves CaT rise time, however it could also increase diastolic [Ca^2+^]_i_, which worsens contraction. These opposing effects should be taken into account when considering blocking NCX. SERCA enhancement in HF is highly effective in reducing t_10-90_, but its depressed activity requires extreme enhancement to restore systolic [Ca^2+^]_i_.

### Comparison with experimental results

In this work the electrophysiological activity of human ventricular myocytes was simulated using the latest human AP model with a detailed formulation of Ca^2+^ dynamics [19], using Ca^2+^ cycling formulated from human experimental data. However, the simulated Ca^2+^ transients and concentrations are generally smaller than those found in the literature, possibly because of the high electrophysiological variability of the experimental data, due in part to the fluorescence-based technique used to measure intracellular Ca^2+^ [5,7,24]. Despite these drawbacks, simulations are able to mimic Ca^2+^ transients and reproduce the abnormalities in Ca^2+^ dynamics observed in failing human hearts. The main alterations are long Ca^2+^ transients of reduced amplitude and SR with less Ca^2+^ content [3,25,26].

Some experimental studies have focused on the main ionic transporters taking part in Ca^2+^ cycling (L-type Ca^2+^ channels, RyR channels, SERCA, and NCX) to improve contraction [14,15,27,28], and many mechanistic details of Ca^2+^ dynamics have been discovered to date. The present work was designed to analyze these mechanisms under different conditions (normal and heart failure) to complement the experimental results and increase our understanding of how they work. From our sensitivity analysis, it can be seen that SERCA plays an important role in modulating Ca^2+^. SERCA’s main function is to remove cytosolic Ca^2+^ and reintroduce it into the SR during diastole for myocardial relaxation. It is therefore reasonable to assume that reducing SERCA activity would increase diastolic [Ca^2+^]_i_, slow the rate of CaT decay, and reduce the SR Ca^2+^ load. SR Ca^2+^ store depletion indirectly reduces Ca^2+^ release and systolic function. In fact, after Ca^2+^ uptake function inhibition, healthy myocytes showed changes in CaT and SR Ca^2+^ content, for example using the use of thapsigargin or TBQ in rats [29–31]. Under failing conditions, we obtained the same alterations in Ca^2+^ indicators, which presented a high sensitivity to SERCA, which highlights the strong effect of SERCA on the rest of the ionic mechanisms affected by HF remodeling. Increased inhibition of the SERCA function resulted in higher alteration in Ca^2+^ dynamics, while the restoration of a normal SR Ca^2+^ uptake showed a significant improvement of all these EP characteristics. Specific pharmacological agents to enhance SERCA activity have not been developed so far, but our results can be compared with those obtained using gene therapy methods. In agreement with our simulations of SERCA variability in normal conditions, transgenic rabbit cardiomyocytes overexpressing SERCA led to an increased magnitude of CaT and SR content compared to the control [32]. However, Morimoto et al. [33] did not find significant changes in SR Ca^2+^ content nor in time to peak in transgenic mice, although SERCA overexpression did increase the CaT peak, which could be related with the insignificant effect on t_10-90_ in normal conditions that we observed in our analysis with enhanced SERCA.

Enhancing SERCA in HF improves cellular Ca^2+^ homeostasis. SERCA overexpression in failing cells restored Ca^2+^ handling in rats [34], as well as in humans [35]. In our simulations, a 60% increase in SERCA activity led to the lower recovery of Ca^2+^ indicators in HF than in normal conditions. Indeed, the depressed activity of the pump in HF is responsible for these discrepancies, and studies should be performed taking into account that different results may be obtained applying the same therapy under normal and failing conditions. Rocchetti et al. [36] observed that istaroxime stimulated SERCA uptake function in failing myocytes, restoring Ca^2+^ levels and, in contrast to our results, the effect on failing myocytes was higher than on the non-failing ones. However, differences between experiments and simulations should be taken with caution. Our 60% SERCA enhancement represents an increase of the downregulated protein in HF whereas istaroxime seems to restore the abnormal SR Ca^+2^ uptake function by targeting secondary molecules interacting with the pump, such as PLB. There might be other sources for discrepancies between our simulation results and Rocchetti et al. experiments, such as species differences (guinea pig vs human myocytes), HF stage, and EP remodeling.

The altered NCX activity in HF also has an impact on Ca^2+^ handling. An increased reverse mode occurs due to [Na^+^]_i_ accumulation, increasing Ca^2+^ influx. At the same time, the upregulated forward mode increases Ca^2+^ efflux, resulting in cellular Ca^2+^ loss and a reduced SR Ca^2+^ load. Therefore, most of the consequences of remodeled NCX worsen Ca^2+^ dynamics and the possible beneficial effect of NCX inhibition has been analyzed in several studies. SEA0400, a NCX inhibitor, did not produce significant changes either in the shape or in the magnitude of the CaT in normal guinea pig or canine myocytes [37–39], although Ozdemir et al. [40] observed an increased CaT peak and SR Ca^2+^ content in pigs. However, positive effects were also observed on Ca^2+^ homeostasis in transgenic animals with NCX overexpression [41,42], as observed in the present simulations. These results suggest a dependency on the animal model and highlight the need for human experimental data, as well as the careful selection of the pharmacological agent. SN-6, another NCX inhibitor, was found to have a negative inotropic action in normal and failing cardiomyocytes, with a greater reduction of SR Ca^2+^ content in HF [43]. In this case, the non-specificity of the blocker and the effect on other currents, such as I_CaL_ can affect the results, so that further experimental studies are required to clarify the effects of NCX inhibition, especially in the failing human myocardium.

The third pathway related to sarcoplasmic Ca^2+^ is its release through RyR channels. In HF, these Ca^2+^ proteins undergo a slightly increased Ca^2+^ sensitivity, which favors the Ca^2+^-induced Ca^2+^ release process. The main problem in HF is that RyRs become leaky and Ca^2+^ is released spontaneously during diastole, typically in the form of Ca^2+^ sparks [44]. As our HF model reproduces the effect of J_leak_, contributing to abnormal Ca^2+^ cycling, its inhibition would thus be beneficial for myocardial contraction. However, in the ORd model, J_leak_ was formulated independently of the RyR channel, representing a continuous Ca^2+^ leakage from the SR through the SERCA pump. In this case, it is not possible to compare our results with the effects of any drug able to alleviate this leak. In our simulation study, sensitivity to RyR channels (J_rel_) was analyzed independently of J_leak_. This ionic mechanism did not have strong effects on Ca^2+^ dynamics in our simulations, unlike most of the published experimental results. Inducing Ca^2+^-leak with caffeine or ryanodine in rats reduced CaT amplitude and changed systolic and diastolic concentrations [30]. Tetracaine, an RyR inhibitor, could reverse these variations and restore normal Ca^2+^ dynamics. Since caffeine decreases SR Ca^2+^ content, increasing the activity of the RyR does not have a positive inotropic effect [45], but the negative effects are not clear.

### Benefits and predictions from systematic sensitivity analyses

Unlike experimental studies, sensitivity analyses are a systematic methodology that allows many parameters to be varied either independently (one at a time) or at the same time, which avoids the difficulty of replicating conditions and allows the control of all the sources of variability that affect experimental results. The selection of the animal species when analyzing cellular electrophysiology is one of the most influential factors. Due to the need for human data and the scarcity of human hearts for experimental purposes, the use of a detailed human model such as the ORd model is extremely useful. Modeling and simulation also allow the assessment of EP characteristics, which cannot be measured experimentally, such as [Ca^2+^] in different cellular compartments. With this approach, the modification of each parameter is quantified, while this cannot always be done accurately in experiments.

The use of sensitivity analyses has been widely extended to study physiological processes providing valuable predictions [16,17]. Other commonly used methods are multivariate analyses, which can provide additional information to that obtained with single parameter analyses [46,47]. However, we chose the simplicity and intuitive interpretation of the results by changing parameters individually, as this is also an accepted method used in this type of study [16,17]. Once the consistency of the sensitivity analyses results have been verified and compared against available experimental data, predictions can be made of how specific EP indicators are modulated by changes in ionic parameters for which data are not available. In agreement with previous studies, I_Kr_ block and I_NaL_ enhancement prolong APD, a situation aggravated under failing conditions [48–50]. The high sensitivity of [Na^+^]_i_ to I_NaK_ and the increase in [Na^+^]_i_ due to the dysfunction of this pump in HF extruding less Na^+^ out of the cell were also as expected [51,52]. This [Na^+^]_i_ accumulation is related to APD shortening when the electrogenic Na^+^/K^+^ pump is inhibited because it increases the NCX reverse mode (outward current) and contributes to cell repolarization [16,17,51,53]. Other modulations of EP characteristics by ionic parameters in HF are less obvious. For instance, blocking I_CaL_ and J_SERCA_ may have negative prolonging effects on APD_30_ which does not occur under normal conditions. In terms of Ca^2+^ characteristics, blocking I_Kr_ prolongs CaTD. The present simulation results predict that several Ca^2+^ indicators are unexpectedly more influenced by changes in I_NCX_ than changes in SERCA in the failing myocyte. Unfortunately, the supposed beneficial blocking of NCX has negative consequences on diastolic [Ca^2+^]_i_ (raising its value), while enhancing SERCA always contributes to improving Ca^2+^ dynamics. These predictions would of course need to be validated using experimental results. The strength of systematic sensitivity analyses is their predictive power and the indications they provide for the design of the required experiments thus contributing to reducing the time and costs involved in drug design.

### Limitations of the study

The limitations of the present study are linked, on the one hand, to the use of a mathematical model for the action potential. Despite its validation using human data, there are still some quantitative differences in intracellular Ca^2+^ levels. Additionally, in some situations, the model does not represent all the ionic mechanisms that are commonly described in the literature as is the case of J_leak_ which, in the ORd model, was formulated only as a backflux of the SERCA pump. However, in other models [21,54,55], J_leak_ is considered as a secondary efflux through the RyR channels, sometimes coexisting with a reverse SERCA flux [44]. For this reason, the analysis of the effects of J_leak_ and J_rel_ should be taken with caution, i.e. accordingly to the model formulation. Also, in our study, J_SERCA_ was considered separately from the J_leak_ term, both of them included in the original ORd J_up_ formulation. Finally, the electrical remodeling associated with HF is not unique [23,56–58], as there are discrepancies and variations regarding some ionic currents due to the data generated in different experimental studies.

The scarcity of available human data has hampered the validation of our results, thus affecting their predictive value, and the wide variations in the existing experimental studies on different species and treatments, also make the validation of the simulations difficult.

## Supporting information

S1 File Supplemental material. Extended Methods and Steady-state conditions.
(DOCX)
