# Supplementary material for: Sensitivity analysis revealing the effect of modulating ionic mechanisms on calcium dynamics in simulated human heart failure
Source: PLoS One. 2017 Nov 8;12(11):e0187739. doi: 10.1371/journal.pone.0187739 (PMC5678731; doi:10.1371/journal.pone.0187739)
Supplement: S1 File — Extended Methods and Steady-state conditions. (DOCX) [file pone.0187739.s001.docx]

Supplemental Material

Extended Methods

The baseline model considered in this investigation is the O’Hara et al. action potential (ORd) [1] with the minor modifications indicated in the main text (ORdmm). All the parameters modulated in the sensitivity analyses are defined and formulated in [1]; some of the equations are detailed in the present section to help the interpretation of the results.

The parameter K_rel,Ca_ appears in the steady-state formulation of J_rel_, i.e. sarcoplasmic reticulum (SR)Ca^2+^ release flux via RyRs (Eq.(1)). K_rel,Ca_ stands for the SR Ca^2+^-dependent activation of J_rel_. As the activation constant of a Hill equation, higher values of K_rel,Ca_ lead to a smaller Ca^+2^ release through RyR. The value of K_rel,Ca_ is reduced in HF, increasing SR Ca^2+^ release [2].

$J_{rel,\infty}=\frac{\alpha_{rel}\cdot(-I_{CaL})}{1+\left( \frac{K_{rel,Ca}}{\left[ {Ca}^{2+} \right]_{jsr}} \right)^{8}}$ (1)

Ca^2+^ leakage refers to a flux of Ca^+2^ leaking from the SR through the SERCA pump, in a reverse passive mode. The driving force only depends on [Ca^2+^]_nrs_ according to Eq.(2):

$J_{leak}=\frac{0.0039375\cdot\left[ {Ca}^{2+} \right]_{nsr}}{15.0}$ (2)

In O’Hara et al’s model [1], the net SERCA pump flux (J_up_) includes the inward active Ca^2+^ uptake (J_SERCA_) and the outward passive leak (J_leak_), as detailed in Eq.(3).

$J_{up}=\left( 1-\emptyset_{up,CaMK} \right)\cdot J_{up,NP}+\emptyset_{up,CaMK}\cdot J_{up,CaMK}- J_{leak}=J_{SERCA}- J_{leak}$ (3)

In the present study, as we were interested in all Ca^2+^ transport mechanisms, we considered J_leak_ and J_SERCA_ independently, to distinguish the passive leak form the active uptake, respectively.

As explained in the Methods section, the sensitivity analysis on HF variability was performed by modifying individually every parameter altered in HF to different degrees of decreased or enhanced activity. These modifications are summed up in Table 1.

Table 1. Individual modulation of heart failure (HF) electrophysiological remodeling. Percentages are indicated with respect to baseline ORdmm model.

| **Ionic parameter** | **No change** | **50% HF** | **HF basic** | **150% HF** |
| --- | --- | --- | --- | --- |
| **I_NaL_** | 100 % | 140 % | 180 % | 220 % |
| **τ_hL_** | 100 % | 140 % | 180 % | 220 % |
| **I_to_** | 100 % | 70 % | 40 % | 10 % |
| **I_K1_** | 100 % | 84 % | 68 % | 52 % |
| **I_NaK_** | 100 % | 85 % | 70 % | 55 % |
| **I_NCX_** | 100 % | 137.5 % | 175 % | 212.5 % |
| **CaMKa** | 100 % | 125 % | 150 % | 175 % |
| **J_SERCA_** | 100 % | 75 % | 50 % | 25 % |
| **J_leak_** | 100 % | 115 % | 130 % | 145 % |
| **K_rel,Ca_** | 100 % | 90 % | 80 % | 70 % |

Steady-state conditions

All the simulations carried out in the sensitivity analyses led to action potential (AP) and Ca^2+^ transient (CaT) curves from which the different quantitative indicators were calculated. Figs A and B show consecutive traces after 1000 s of simulation, the achievement of the steady state, and the most important differences after specific ionic modulation.


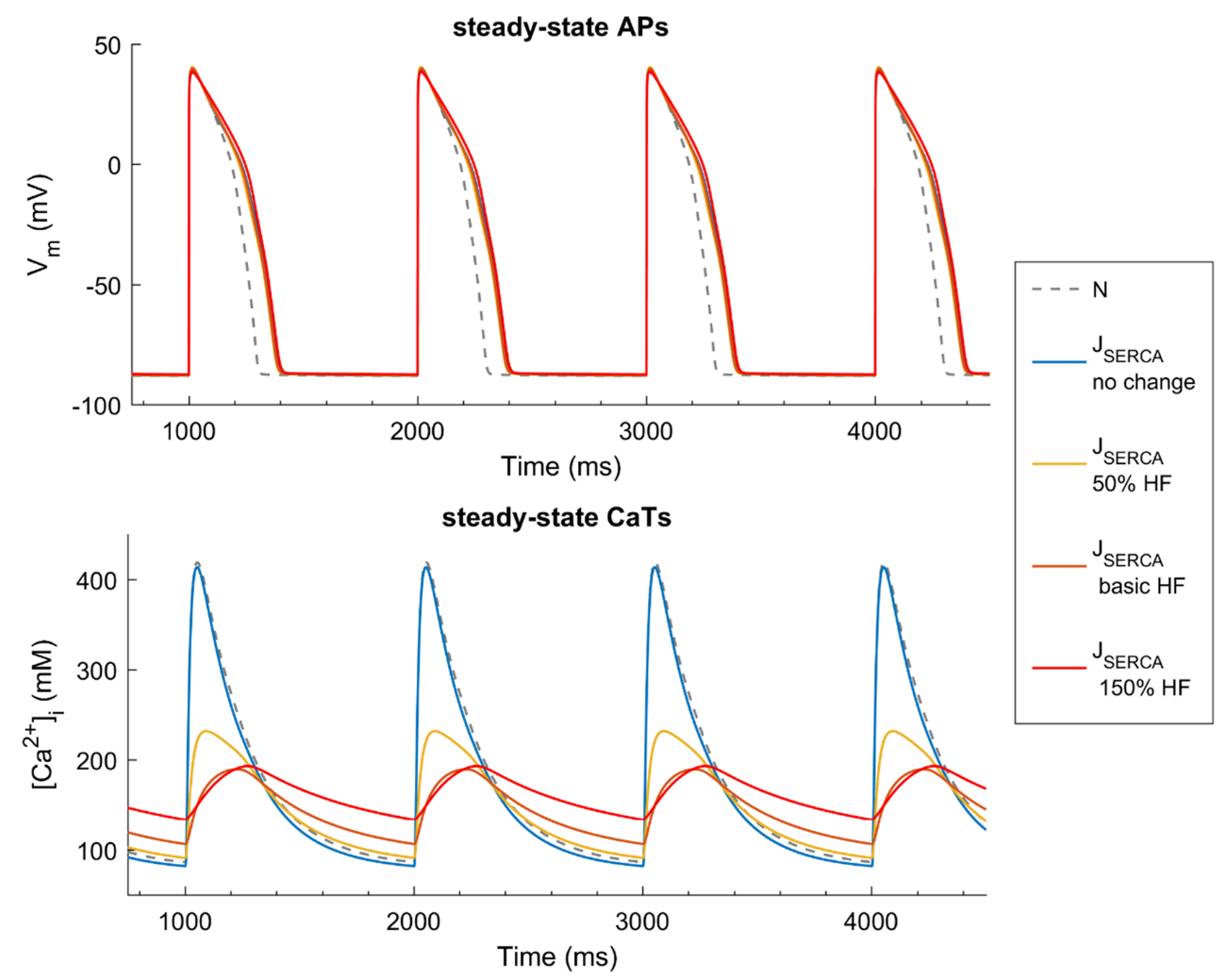


Fig A. Steady – state time course of the last APs and CaTs after 1000 s of simulation in the heart failure (HF) sensitivity analysis. Modification of J_SERCA_ parameter.


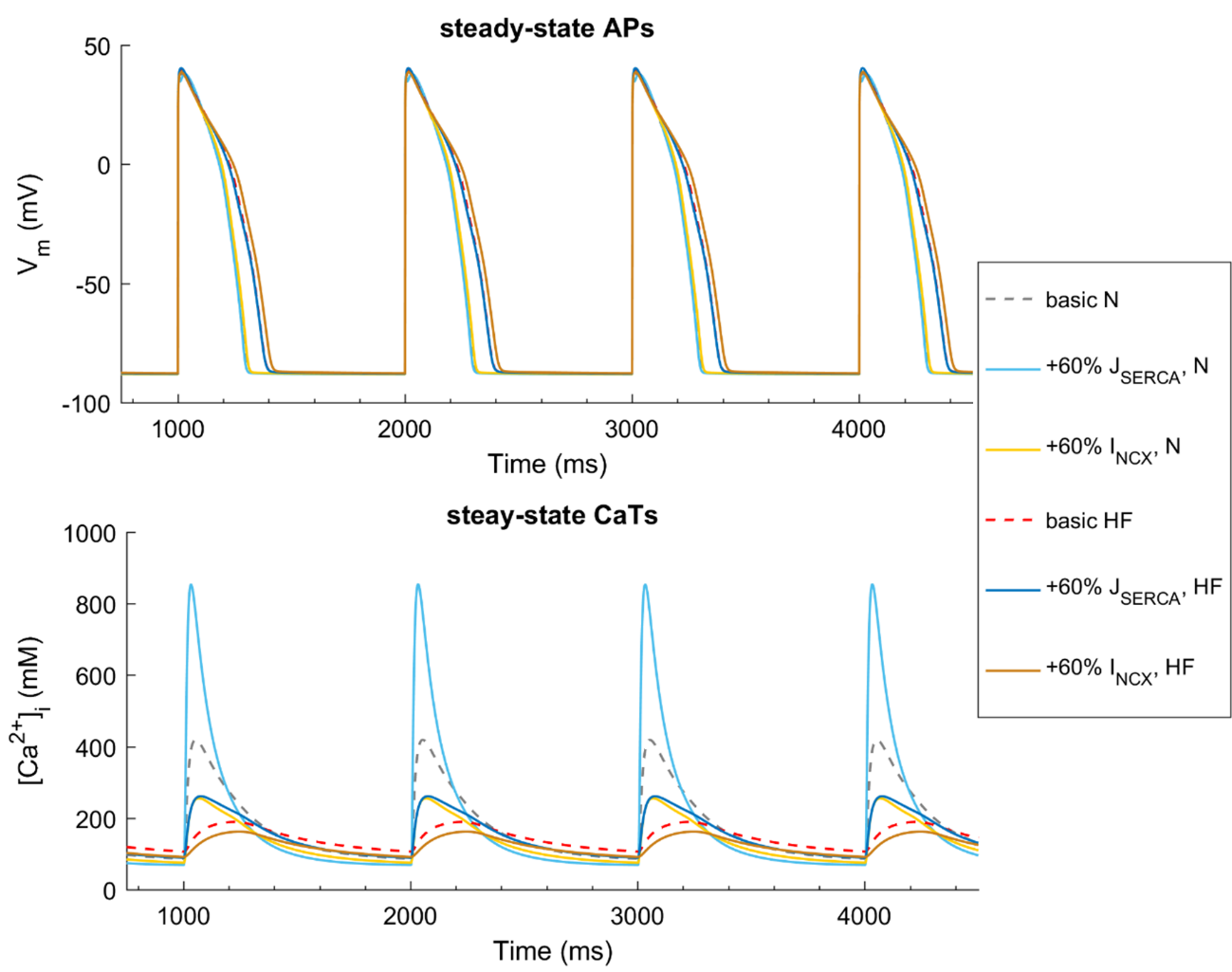


Fig B. Steady – state time course of the last APs and CaTs after 1000 s of simulation in the sensitivity analysis of ‘drug’ effects. Modulation of J_SERCA_ and I_NCX_ parameters in normal (N) and heart failure (HF).

References

1. O’Hara T, Virág L, Varró A, Rudy Y. Simulation of the undiseased human cardiac ventricular action potential: model formulation and experimental validation. PLoS Comput Biol. 2011;7(5):e1002061.

2. George CH. Sarcoplasmic reticulum Ca2+ leak in heart failure: Mere observation or functional relevance? Cardiovasc Res. 2008;77(2):302–14.
